# Hemispheric Differences in Relational Reasoning: Novel Insights Based on an Old Technique

**DOI:** 10.3389/fnhum.2015.00055

**Published:** 2015-02-09

**Authors:** Michael S. Vendetti, Elizabeth L. Johnson, Connor J. Lemos, Silvia A. Bunge

**Affiliations:** ^1^Helen Wills Neuroscience Institute, University of California at Berkeley, Berkeley, CA, USA; ^2^Department of Psychology, University of California at Berkeley, Berkeley, CA, USA

**Keywords:** reasoning, hemispheric specialization, deductive, transitive inference

## Abstract

Relational reasoning, or the ability to integrate multiple mental relations to arrive at a logical conclusion, is a critical component of higher cognition. A bilateral brain network involving lateral prefrontal and parietal cortices has been consistently implicated in relational reasoning. Some data suggest a preferential role for the left hemisphere in this form of reasoning, whereas others suggest that the two hemispheres make important contributions. To test for a hemispheric asymmetry in relational reasoning, we made use of an old technique known as visual half-field stimulus presentation to manipulate whether stimuli were presented briefly to one hemisphere or the other. Across two experiments, 54 neurologically healthy young adults performed a visuospatial transitive inference task. Pairs of colored shapes were presented rapidly in either the left or right visual hemifield as participants maintained central fixation, thereby isolating initial encoding to the contralateral hemisphere. We observed a left-hemisphere advantage for encoding a series of ordered visuospatial relations, but both hemispheres contributed equally to task performance when the relations were presented out of order. To our knowledge, this is the first study to reveal hemispheric differences in relational encoding in the intact brain. We discuss these findings in the context of a rich literature on hemispheric asymmetries in cognition.

## Introduction

Relational reasoning is a cognitive process that requires the joint consideration of relations in order to generate an inference to support a conclusion. Although there is a wide range of theoretical models for relational reasoning (for review, see Goodwin and Johnson-Laird, 2005; Knowlton et al., 2012), all of these models present relational reasoning as a unitary system. However, work from neuropsychological and neuroimaging literatures indicates that some cognitive functions may be supported by multiple, redundant systems in the brain (Roser and Gazzaniga, 2004; Marinsek et al., 2014). Here, we sought to test whether one hemisphere displays an advantage over the other during relational encoding, or whether this function can be carried out equally well by each hemisphere.

Hints of a possible left-hemisphere advantage in relational reasoning have emerged over the course of a number of neuroimaging experiments (e.g., Goel and Dolan, 2004; Green et al., 2006; Bunge et al., 2009; Wendelken et al., 2011). Importantly, similar patterns have been observed for tasks involving either verbal or non-linguistic/pictorial stimuli, suggesting that the observed differences are not entirely stimulus-driven and do not completely overlap with regions supporting language (Monti and Osherson, 2012). However, the conclusions we can draw from these fMRI studies about lateralization of function are limited in several ways. Namely, brain imaging provides correlational rather than causal evidence, and results depend on the specific contrasts used as well as the choice of statistical threshold. All of these factors can mask whether both hemispheres are indicated as being involved in a particular task, and thus, any conclusions about localization should converge with experimental findings using multiple approaches.

The neuropsychological literature also hints at possible hemispheric differences in contributions to reasoning. Much of the early work investigating differential hemispheric contributions to cognitive function came from work on split-brain patients (e.g., Sperry et al., 1969). These studies indicated an improved ability for hypothesis testing during problem solving in the left relative to the right hemisphere (LeDoux et al., 1977) and has led to the idea of the left hemisphere being an “interpreter” of events – i.e., the hemisphere with a major role of integrating newly acquired perceived information with previously constructed theories (Gazzaniga, 2000; Marinsek et al., 2014).

Following the seminal work of Gazzaniga et al. (1962) indicating how cognitive function differed in the two hemispheres following sectioning of the commissures, hemispheric asymmetries in cognition have alternately been characterized as a dichotomy between local and global (van Kleeck, 1989), categorical and coordinate (Kosslyn, 1987; van der Ham et al., 2014), or serial and parallel (e.g., Cohen, 1973) processes (for review, see Bradshaw and Nettleton, 1981). In the present study, we did not set out to evaluate these competing accounts of hemispheric specialization; rather, we sought to characterize the contribution of each hemisphere to performance of a relational reasoning task adapted from one used in a prior fMRI study from our group (Wendelken and Bunge, 2010).

There is not a consistent pattern relating relational reasoning ability to damage in a particular hemisphere. Neuropsychological work on relational reasoning has demonstrated the necessity of prefrontal and posterior parietal regions during transitive inference (Waltz et al., 1999; Krawczyk et al., 2008; Waechter et al., 2012), analogical reasoning (Morrison et al., 2004; Krawczyk et al., 2008), and matrix reasoning (Baldo et al., 2010; Woolgar et al., 2010). Additionally, studies employing voxel-based lesion symptom mapping to investigate relationships between patterns of brain damage and resulting cognitive deficits in fluid intelligence (Barbey et al., 2014) have suggested that damage to the right hemisphere plays a more critical role. However, Baldo et al. (2010) demonstrated that patients who have incurred strokes in the left hemisphere have been shown to also have significant deficits in a visuospatial relational reasoning task; therefore, more research is needed to provide a better understanding of each hemisphere’s role in relational reasoning.

We designed the current study to test the role of each hemisphere in relational encoding through the use of a visual half-field stimulus presentation procedure. This paradigm was originally developed for use in split-brain patients, who have either minimal or no connection between the two hemispheres (e.g., Gazzaniga et al., 1962). Here, our participants were healthy adults whose hemispheres are presumed to interact closely in the coordination of task performance (Weissman and Banich, 2000). Nevertheless, we sought to test for differences in response times and/or accuracy when relational information is *initially encoded* by the left or the right hemisphere. This visual half-field stimulus presentation procedure allowed us to test whether left and right hemispheres differentially support relational encoding.

**Figure 1:**
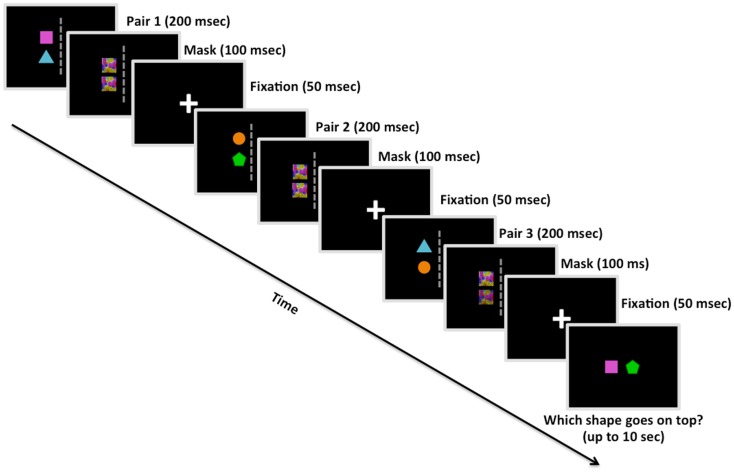
**Example trial from Study 2 (including the visual mask)**. Participants were shown three pairs of colored shapes. Following each pair, participants were shown a visual mask overlaying the previous shapes, and then a fixation cross. After the third pair was presented in a given trial, participants had up to 10 s to decide the correct linear order of two shapes based on the spatial relationships observed among the pairs. This is an example of a reordered trial, in which participants would presumably have to manipulate their memory of the pairs in order to deduce that the square goes on top of the pentagon. Study 1 was similar in design except for the absence of the visual mask presentations.

In the present study, we used a transitive inference task adapted from an fMRI task that we have used previously (Wendelken and Bunge, 2010). When reasoning using transitive inference, the logical conclusion is deduced through transferring relational inferences among terms expressed in the premises (e.g., if A > B and B > C, then A must be greater than C). On this task, shown in Figure 1, participants view a new set of relations on every trial and are expected to integrate them in working memory. There has been a rich literature on this form of reasoning (e.g., Halford, 1984; Cohen et al., 1997; Andrews and Halford, 1998; Greene et al., 2001). Importantly, this form of relational reasoning bears only a passing resemblance to transitive inference paradigms that involve learning paired associations over many trials (e.g., Acuna et al., 2002; Zalesak and Heckers, 2009; Koscik and Tranel, 2012; for discussion, see Wendelken and Bunge, 2010). The major difference between our transitive inference paradigm and those based on learning paired associations is that our task does not rely on remembering associations to be transferred; instead, participants must infer the spatial relationship based on the relations from the most recent trial only. Having to perform this inference anew each trial reduces any tendency to assume an object-order relationship when attempting to solve the task.

Inspired by neuropsychological research demonstrating that prefrontal patients have difficulty with transitive inference when the relations are presented out of order (e.g., “Sam is taller than Roy,” “James is taller than Sam”; Waltz et al., 1999; Krawczyk et al., 2008), we manipulated the sequence of presentation of the three relations. On half of the trials, the relations were *ordered* (A > B; B > C; C > D), and on the other half, they were *reordered* (A > B; C > D; B > C *or* C > D, A > B, B > C). We hypothesized that manipulating encoding in this manner would have an influence on the downstream integration process, and sought to test for hemispheric differences in performance on trials whose relations could be integrated readily (*ordered* trials) and those that could not (*reordered* trials).

## Materials and Methods

### Participants

*Experiment 1:* Twenty-three healthy adults (14 female, aged 18–34 years; $\bar{X}\pm \text{SD}$ age, 22 ±3.08 years). *Experiment 2:* Thirty-one healthy adults (24 female, aged 18–25 years; $\bar{X}\pm \text{SD}$ age, 20 ±1.80 years). All participants attended the University of California, Berkeley, and participated in either Experiment 1 or 2 for partial fulfillment of a course requirement. All participants had normal or corrected-to-normal vision, were right-handed, and were fluent in English. Participants had no reported history of neurological or psychiatric disorders. All participants gave their informed consent to participate in the study, which was approved by the Committee for Protection of Human Subjects at the University of California, Berkeley.

### Design

We ran two studies with a similar design except for the addition of brief visual masks immediately following presentation of each object pair (100 ms) and an additional 48 trials, both of which were implemented in Experiment 2. We chose to insert the visual masks in Experiment 2 to reduce any after-image perceptual influences on decision making, in effect making the participant’s deduction solely based on information stored and manipulated in working memory (Kim and Blake, 2005). The task designs were identical with the exception of these additions in Experiment 2; therefore, all of the information below applied to both studies unless explicitly stated. The stimulus set consisted of four colored shapes: blue triangle, orange circle, green pentagon, and pink square. On each trial, three sets of relations – pairs of shapes arranged vertically, with one colored shape positioned directly above another colored shape – were presented in sequence (Figure 1). One-third of the transitive inference trials involved *ordered* problems, in which the source relations were presented in order (e.g., A > B, B > C, C > D; A – D?); the other two-thirds involved *reordered* problems, in which the middle relation was presented last (e.g., A > B, C > D, B > C; A – D? *or* C > D, A > B, B > C; A – D?). Placing the middle relation last instead of the final relation of the sequence assured that participants could not rely on simple memory for the most recent pair when making their decision.

Prior to the onset of each trial, white arrows appeared coming from the four corners of the screen for 400 ms in order to direct eye gaze to the center of the screen. Trials began with a white central fixation cross displayed on screen for 50 ms. Each pair of shapes was presented in the left or right visual hemifield for 200 ms, followed by a visual mask for 100 ms (Experiment 2 only) and a central fixation inter-stimulus interval (ISI) for 50 ms, and then a different pair of shapes in either the same or opposite visual hemifield for 200 ms. After being shown three pairs individually, participants were asked to deduce the correct linear order of two items (e.g., square and pentagon) based on the spatial relations presented in the sequence of object pairs (e.g., square above triangle, triangle above circle, and circle above pentagon). Participants had ≤10 s to make their decision regarding the correct linear order of two colored shapes (i.e., which of the two objects would be on top following the spatial relations represented in the trial).

### Procedure

Participants placed their heads in a chinrest affixed at arm’s length from the screen, and were instructed to maintain their gaze on a central fixation cross. Vertical pairs of shapes were displayed between 4° and 6° of visual angle from central fixation (Buschman et al., 2011).

In Experiment 1, the task included 96 trials total: 24 in which all three shape pairs were presented to the left hemisphere (LLL), 24 in which they were presented to the right hemisphere (RRR), 24 in which they were presented to alternating hemispheres (12 LRL and 12 RLR trials), and 24 in which they were presented to opposite hemispheres but did not alternate (12 LRR and 12 RLL trials). The LRL, RLR, LRR, and RLL trials were inserted so that participants could not reliably predict where the second and third pairs would be presented. Experiment 2 included an additional 48 trials, but the balance of trial types was consistent with Experiment 1. Trials were evenly counterbalanced by hemispheric presentation and ordering condition, and the trial order was fully randomized.

The final prompt displayed two shapes next to each other and participants were instructed to indicate via key press which shape should “go on top” based on the information in the three pairs of relations. The “z” key corresponded to the shape on the left and the “?/” key to the shape on the right; participants were instructed to keep their left hand on the “z” key and right hand on the “?/” key throughout the trials. In half the trials, the correct answer appeared on the left and half on the right. Participants were given a short break at the mid-point of the task. Experiment 2 contained a third block of trials, so participants were given a second break.

## Results

### Fully lateralized trials

**Figure 2:**
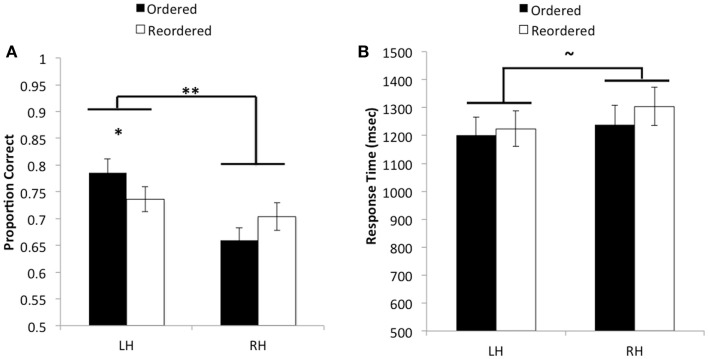
**(A)** Average proportion correct as a function of hemisphere and ordering condition. A significant interaction was found such that when pairs of objects were presented in order, performance was significantly better when information was initially presented to the left versus the right hemisphere. However, no reliable difference was observed between hemispheres when pairs needed to be reordered in memory. Additionally, an overall main effect was found indicating that accuracy improved when pairs were initially encoded by the left hemisphere as opposed to the right hemisphere. **(B)** Average response time in milliseconds as a function of hemisphere and ordering condition, for correct trials. No reliable differences were observed for response time. **p < 0.01.

We first investigated whether the small differences in task design between Experiments 1 and 2 would lead to any reliable differences in the results. A three-way mixed effects analysis of variance (ANOVA) with experiment number as the between-subjects variable, and hemispheric presentation (LH versus RH) and ordering condition (ordered versus reordered) as within-subjects variables indicated neither a main effect of experiment nor any interaction with other factors, *F’s* < 1, *p’s* > 0.54. Thus, all subsequent reported effects were generated from models collapsing across studies^1^. We analyzed accuracy and response time data in separate two-way repeated measures ANOVAs, with hemispheric presentation and ordering condition as within-subjects factors. In this first section, we discuss only those trials that were solely presented to the left or right hemisphere. Behavioral results are presented in Figure 2.

The ANOVA revealed a significant main effect of hemisphere on accuracy, *F*(1, 53) = 27.15, *MSE* = 0.012, *p* < 0.01, $\eta {\;}_{\mathrm{partial}}^{2}=0.34$, such that participants performed better when relational information in the reasoning problem was initially encoded by the left hemisphere ($\bar{X}$ = 0.76, *SD* = 0.17) as compared to the right hemisphere ($\bar{X}$ = 0.68, *SD* = 0.16). A significant interaction between hemispheric presentation and ordering condition was also observed, *F*(1, 53) = 8.2, *MSE* = 0.013, *p* < 0.01, $\eta {\;}_{\mathrm{partial}}^{2}=0.13$. *Post hoc t*-tests using Bonferroni correction showed that participants were significantly more accurate when ordered pairs were presented to the left hemisphere ($\bar{X}$ = 0.79, *SD* = 0.19) as compared to the right hemisphere ($\bar{X}$ = 0.66, *SD* = 0.16), *t*(53) = 6.02, *p* < 0.001, $\eta {\;}_{\mathrm{partial}}^{2}=0.41$. By contrast, no significant differences were found in accuracy between the left hemisphere ($\bar{X}$ = 0.74, *SD* = 0.17) and right hemisphere ($\bar{X}$ = 0.70, *SD* = 0.19) on reordered trials, *t*(53) = 1.51, *p* > 0.13, $\eta {\;}_{\mathrm{partial}}^{2}=0.04$. We could also describe this interaction by looking at differences between trial types within each hemisphere. Although neither of these comparisons passed Bonferroni correction, in the left hemisphere, performance on ordered trials was better than on reordered trials, whereas the opposite was true in the right hemisphere. These results suggest that, although performance was best when stimuli were presented in order to the left hemisphere, both hemispheres performed similarly when relations were not presented in an order that is conducive to integration before solving the transitive inference problem.

When including response times from correctly performed trials as the dependent variable, the ANOVA produced a marginally significant effect of hemispheric presentation, such that participants were faster to produce the correct decision on trials that were presented to the left hemisphere ($\bar{X}$ = 1218.41, *SD* = 433.56) as compared to the right hemisphere ($\bar{X}$ = 1273.10, *SD* = 448.26), *F*(1, 53) = 3.93, *MSE* = 41115.21, *p* = 0.053, $\eta {\;}_{\mathrm{partial}}^{2}=0.07$. No other effects in relation to response time were found to be statistically significant, *F’s* < 1.26, *p’s* > 0.26. These results suggest that the left-hemisphere boost in performance was not due to a speed-accuracy tradeoff; rather, when object pairs were presented to the left hemisphere, participants tended to respond faster than they would have if information had been presented to the right hemisphere.

### All trials

**Figure 3:**
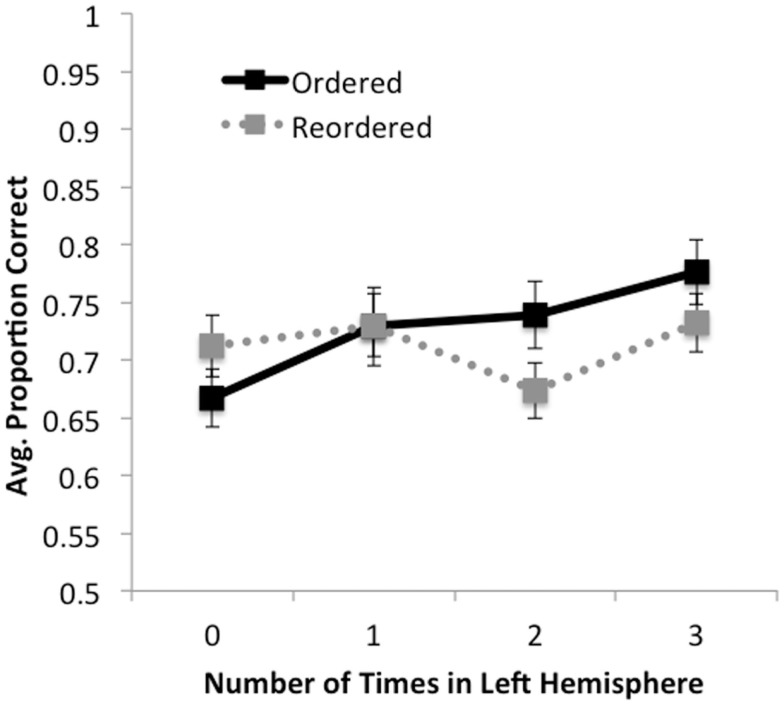
**Accuracy as a function of ordering condition and number of times premise was presented in the left hemisphere (0, 1, 2, 3)**. For ordered trials, accuracy increased monotonically with the number of times a premise was presented in the left hemisphere. For reordered trials, a simple pattern was not observed; rather, accuracy decreased when premises were presented in the left hemisphere two times (i.e., on LRL and RLL trials) relative to one or three times. No effects were observed for response times.

In this section, we describe analyses investigating performance across both fully lateralized and mixed hemisphere trials (Figure 3). We ran 4 × 2 repeated measures ANOVAs with number of times in the left hemisphere (0, 1, 2, 3) and order (*ordered* versus *reordered*) as within-subject factors, predicting accuracy and response time scores in separate models.

No significant effects were found for response times, *F’s* < 1.8, *p’s* > 0.18. In terms of accuracy, we found a significant main effect of number of times in the left hemisphere, *F*(3,159) = 8.79, *MSE* = 0.013, *p* < 0.001, $\eta {\;}_{\mathrm{partial}}^{2}=0.14$, such that greater accuracy was observed the more often premises were presented in the left hemisphere. We also observed a trend for the effect of order, such that accuracy on ordered trials ($\bar{X}$ = 0.74, *SD* = 0.16) was marginally higher than on reordered trials ($\bar{X}$ = 0.72, *SD* = 0.15), *F*(1,53) = 3.45, *MSE* = 0.017, *p* < 0.07, $\eta {\;}_{\mathrm{partial}}^{2}=0.06$. We observed a significant interaction between number of times in the left hemisphere by order, *F*(3, 159) = 5.55, *MSE* = 0.013, *p* < 0.001, $\eta {\;}_{\mathrm{partial}}^{2}=0.1$. We found that for ordered trials there was a significant monotonic increase in accuracy as premises were presented to the left hemisphere, *F*(1, 53) = 38.11, *MSE* = 0.011, *p* < 0.001, $\eta {\;}_{\mathrm{partial}}^{2}=0.42$. For reordered trials, no such linear trend was observed, *F*(1, 53) < 1, *p* > 0.5. These results suggest that when information is already ordered, increases in accuracy can be significantly predicted by how many times the premises are presented in the left hemisphere, and support our finding that participants performed better when ordered trials were presented only to the left hemisphere than to the right.

### Follow-up analyses

In testing for hemispheric differences in performance on this transitive inference task, we sought to ensure that participants were performing this task in the manner expected. When three relations are presented in order, it is possible to produce the correct response even without integrating multiple relations (Bryant and Trabasso, 1971). In our design, this simpler, non-integrative strategy could be undertaken by paying attention only to the top item in the first premise rather than encoding all premises and integrating the relations between them. If participants were to take this strategy, they would be expected to achieve roughly 100% accuracy on ordered trials, but only around 50% accuracy on reordered trials (because the first item of the first premise only appeared in the final prompt on two-thirds of the trials). Six out of 54 participants exhibited a pattern consistent with the use of this strategy. The findings reported here hold even when excluding these six participants.

## Discussion

Inspired by findings from the neuroimaging and neuropsychological literatures, we tested whether healthy young adults’ performance on a reasoning task would differ on whether the stimuli were presented to the left or right hemisphere. By designing a transitive inference task with visual half-field stimulus presentation, we were able to show differences in reasoning performance as a function of the hemisphere that initially encoded the sets of visuospatial relations. Given that the two hemispheres communicate freely in the intact brain, we had expected only modest differences in response times for left- versus right-hemifield stimulus presentation. As such, we were surprised by the magnitude of the behavioral difference elicited by visual half-field presentation in this study, with an average difference in accuracy of 11% between left-lateralized and right-lateralized ordered trials. Although claims of inter-hemispheric differences in cognition have been made for many years (Gazzaniga et al., 1962; Cohen, 1973), our study is the first to demonstrate hemispheric differences in relational encoding in neurologically intact participants.

Although task performance (i.e., accuracy) improved overall when participants encoded the visuospatial relations in the left hemisphere, this effect was driven by performance on the ordered trials. That is, we observed a left-hemisphere advantage when the relations were ordered linearly and, therefore, could be integrated directly, but not when it was necessary to rearrange the relations before integrating them. For right-hemisphere trials, participants did not show the predicted pattern of worse performance for reordered versus ordered trials. This pattern was unexpected, and warrants further investigation. Surprisingly, given that reordered trials are hypothesized to require additional processing relative to ordered trials (Waltz et al., 1999; Krawczyk et al., 2008), left-hemisphere encoding of *reordered* relations was superior even to right-hemisphere encoding of *ordered* relations. These results suggest that the left hemisphere excels at relational encoding.

The present results fit well with neuroimaging studies that have pointed toward a left-hemisphere specialization in relational reasoning (Wendelken et al., 2008; Bunge et al., 2009; Green et al., 2010). In light of these findings, it is interesting to consider a recent resting-state functional connectivity study showing that the left-hemisphere interacts more exclusively with itself, whereas the right hemisphere demonstrates connectivity patterns associated with both hemispheres (Gotts et al., 2013). This result suggests that the left hemisphere may operate independently, whereas the right hemisphere functions, at least partly, with assistance from the left hemisphere. Given these findings, we would predict a left-hemisphere advantage if relational encoding hinges more on intra-hemispheric interactions, and indeed this prediction was supported by our analysis including the mixed trials.

### A left-hemisphere advantage for relational encoding

The behavioral improvement observed in our study does not indicate that the right hemisphere cannot encode relational information, but rather suggests that relational encoding may be processed more effectively in the left hemisphere. Although the stimuli were visuospatial in nature, they nonetheless were easily identifiable verbally (e.g., circle, square, pentagon). Given how quickly premises were presented, it does not seem feasible that very many participants would have had enough time to verbally label objects while they solved the task; however, we cannot conclusively rule out this possibility. The present study establishes a paradigm that could be used for further examination of the necessity of verbal labeling for relational reasoning.

Numerous dichotomies have been used to explain hemispheric asymmetries in cognitive functioning (Bradshaw and Nettleton, 1981), and so we do not claim that the left-hemisphere advantage observed in our study is unique to relational encoding, *per se*. Beyond the verbal/non-verbal distinction (Gazzaniga et al., 1962), other theories have focused on local versus global (van Kleeck, 1989), serial versus parallel (Cohen, 1973), holistic versus analytic (Nebes, 1978; Cooper and Wojan, 2000), categorical versus coordinate (Kosslyn, 1987), or syntactical versus intuitive/“gist” (Bogen, 1975; Phelps and Gazzaniga, 1992) processing, to name a few. Such dichotomies are useful in that they demonstrate how a higher level cognitive task such as reasoning might be represented as a combination of lower order cognitive processes. Our transitive inference task could be construed as being syntactical, serial, and analytic, and previous work focusing on these distinctions has consistently demonstrated a left-hemispheric specialization (for review, see Bradshaw and Nettleton, 1981). Additionally, encoding spatial relations in the premises categorically (e.g., identifying the square as above the triangle) would also fit with previous work demonstrating a left-hemispheric advantage for categorical encoding of spatial relations (Kosslyn, 1987; van der Ham et al., 2012).

## Conclusion and Future Directions

Our results shed light on cognitive theories of relational reasoning, as they provide evidence for differential processing of relations by the two hemispheres. Specifically, we found that participants performed better on our transitive inference task when the premises were presented to the left hemisphere. This effect was driven by an interaction such that there was a greater difference in performance when the premises were ordered than when participants presumably had to reorder the premises before making their conclusion. Theories describing a unitary mechanism of relational reasoning (e.g., Hummel and Holyoak, 2003; Goodwin and Johnson-Laird, 2005) may need to incorporate multiple components in order to fully represent interhemispheric differences used during relational reasoning.

The present results are consistent with theoretical predictions concerning hemispheric specialization of cognitive functions. Specifically, participants are expected to perform better when information is presented to the left hemisphere for tasks that could be solved using a stepwise and analytical strategy. Our findings extend previous work given that our transitive inference task not only exemplifies these types of strategies but also relies on the comparison of relational information between premises in order to arrive at a solution.

These behavioral results warrant further investigation with neuroscientific techniques. First, functional imaging techniques could be used to measure the dynamic interplay between hemispheres during performance of this lateralized transitive inference task. Second, transcranial direct current stimulation could be used to increase or reduce cortical excitability within a hemisphere and test whether relational reasoning performance in each hemisphere changes as a function of cortical excitability (Nitsche and Paulus, 2001; Ardolino et al., 2005). Finally, patients with unilateral brain injuries could be tested on this lateralized task to assess whether relational encoding is primarily a left-hemisphere function, or whether the right hemisphere could specialize in this function after left-hemisphere damage. Thus, reapplying this well-established stimulus presentation procedure in these multiple contexts will help us to better understand the underlying mechanisms required for processing relational information during reasoning.

## Conflict of Interest Statement

The authors declare that the research was conducted in the absence of any commercial or financial relationships that could be construed as a potential conflict of interest.

## Footnotes

^1^Including gender as a factor in the full model, we found that the males in this study were more accurate than the females. Given the large gender imbalance in our relatively small sample, this result should not be over-interpreted. Notably, both males and females exhibited higher accuracy when the relations were presented to the left hemisphere than to the right hemisphere.
